# Remarks on Sources and Varieties of Vaccine Virus

**Published:** 1881-05

**Authors:** Oscar C. DeWolf

**Affiliations:** Health Officer of Chicago


### Article II.

### Remarks on Sources and Varieties of Vaccine Virus. By Oscar C. DeWolf, M. D., Health Officer of Chicago.

In the January number of the *N. Y. Medical Abstract* is a brief review of a late discussion on vaccination by the Medical Society of Kings County.

Dr. J. H. H. Burge, in closing the discussion, makes the following inquiry: "What virus have we now in use in this country?" And he answers, "Probably six or seven kinds:

"1. We have, I hope, though I don't know where to obtain it, virus direct from the natural vaccination.

"2. We have humanized virus from the natural bovine vesicle.

"3. A virus resulting from humanized virus introduced again to the system of the cow, and brought back to man.

"4. Probably a virus resulting from inoculation of the cow with small-pox matter.

"5. It is not impossible that we have a virus from other vesicular diseases in the cow resembling vaccinia.

"6. We have the calf-to-calf virus, originally from the true natural kine-pox, and descending through a long line of induced cases of vaccinia."

If this statement is true it is a grave impeachment of vaccine cultivators in this country, and the subject is of so much importance that I trespass on the courtesy of this society to briefly present the result of such facts as I have been able to gather.

The cultivation of vaccine matter by continuous calf-to-calf inoculation from which animals, as an endless source of supply, virus could readily be obtained, was practically unknown to the profession before the year 1866. Long previous to this date, however, animal vaccination had been practiced in Italy, where Troja, of Naples, conceived the idea of taking the virus from the arm of a child and vaccinating the heifer, which was perpetuated through a series of animals. This was simply retro-vaccination, *i. e.*, implanting upon the animal the same vaccine disease from the child which Jenner had methodically transferred to this child from the animal.

To this date, about 1803, we have no knowledge of the so-called spontaneous appearance of cow-pox in any animal other than those observed by Jenner in England late in the previous century.

Negri, the neighbor and successor of Troja, followed in the steps of his master, vaccinating such people as would submit to the operation, until about the year 1820 he discovered a case of natural cow-pox in Calabria, and thereafter propagated entirely from this source. For a subsequent period of 44 years animal vaccination was confined entirely to Italy.

In 1864, Dr. Palasciano, of Naples, addressed the Medical Congress, assembled at Lyons, on the subject of animal vaccination as practiced by his townsman Negri. So impressed was one of his listeners, Dr. Lanoix, that he visited Naples immediately to study animal vaccination under Negri. On his return to France he brought with him a calf nine months old vaccinated by his teacher. Stopping at the railroad station at Lyons only long enough to permit Dr. Diday to take sufficient matter from the heifer to vaccinate several children, he hastened with his charge to Paris. Here he attempted for nearly two years to interest the profession and the public in his work, with only a moderate success.

There was a great deal of doubt expressed in regard to this Neapolitan stock of vaccine virus. In spite of all the assertions to the contrary the general opinion declared that it was a case of retro-vaccination, and not true heifer-transmitted cow-pox.

And now appears the famous heifer from Beaugency, which has revolutionized public and professional opinion touching animal vaccination.

It is idle to speak of any specific infection as originating spontaneously, yet in referring to this heifer, historians treat her disease as appearing spontaneously, and we continue the fiction.

A woman (Dronin) living in the town of Beaugency, in the department of Loiret, fifteen miles from Orleans, visited her yard as usual, on the morning of March 28, 1866, to milk her cow. The heifer was thirty months old, white, of the Breton breed, and had dropped her calf four months previous. She was astonished to find her less gentle than usual, and upon examination discovered some remarkable vesicles on the skin of the udder at the base of the teats. A veterinary surgeon recognized at once the character of the eruption, and M. Depaul, the director of the government vaccine establishment in Paris, was notified. He at once visited Beaugency, bought a fine calf which he vaccinated from the white heifer, and departed for Paris with his prize.

Depaul's calf developed twenty-seven fine vesicles, and thus was inaugurated the true and undoubted method of animal vaccination. Heifer-to-heifer vaccination was continued by Depaul, and his virus was rapidly distributed throughout France. In June, 1870, Dr. Henry A. Martin, of Roxbury, Mass., dispatched an agent to Paris to obtain virus from this source. Depaul supplied him with matter from the 258th, 259th and 260th of his continuous series from the heifer of Beaugency.

This agent reached Boston on the 23d of September, and on the same day Martin vaccinated three heifers, on the next day two, and two each day thereafter until ten were vaccinated and the supply exhausted. For six succeeding years Martin's stock was exclusively used in this country.

Dr. Frank P. Foster, of New York, in charge of the vaccine dispensary, commenced the propagation of virus from Martin's heifers in November, 1870.

Dr. E. L. Griffin, of Fond-du-Lac, Wis., established his stables in November, 1872, using the same stock. The two or three other establishments in New England, aside from Martin's, obtained their virus from him. Within the last three years other stocks have been introduced.

Dr. Griffin, of Wisconsin, lost by accident his original stock in 1880. He at once procured a new virus from the service of the government director of vaccine at Brussels. The pedigree of this stock I cannot trace further than this, that Dr. Warlomont, the director of the Belgian establishment, introduced into his stables in 1868 the "Esneaux lymph," so called from a neighborhood in which a case of spontaneous heifer disease was found. Dr. Warlomont is the most prominent propagator of vaccine virus in Europe, and the prophylactic power of this Belgian stock is probably second to none. Dr. Pottet, of Cleveland, is propagating from Griffin's recent stock.

The Jenner vaccine farm at Chambersburg, Pa., claims to be propagating the Beaugency stock, imported from France. This claim is doubted by reliable gentlemen with whom I have corresponded in this country and Europe. The Franco-German war so interfered with all industries, that among other results the service of heifer vaccine was totally abandoned in Paris during the siege, and the Beaugency stock was absolutely lost in

France. This Pennsylvania firm being of recent date, I presume to be using the Belgian stock.

Dr. W. E. Griffiths, of Brooklyn, N. Y., commenced in 1878 the propagation of virus from a spontaneous heifer disease discovered in Central New York. I do not obtain a satisfactory pedigree of this stock, but it seems to be reliable, for it has established itself as a vigorous prophylactic.

One propagator on the Pacific Coast, one in Wisconsin, and three in the Middle States, other than those previously mentioned, are cultivating virus from Martin's or Griffin's stock. It remains only to refer to attempts to variolate the cow, and the probability of any such virus being found in this country. There is no doubt that Jenner regarded small-pox in man, and cow-pox in the cow, as identical diseases, or manifestations of a single cause. There is no evidence, however, that he ever attempted the transferal of variolous matter from a man to a cow. Jenner's hypothesis was denied by many of his contemporaries and successors, but Dr. Martin in his very able report on animal vaccination to the American Medical Association, in 1877, says: "Accumulated experience, some of which is quite recent, seems very decidedly to confirm this wonderful guess." Many experimentors have attempted this difficult feat and have generally failed.

Ceely and Badcock, of England, in 1839 and 1840, inoculated 600 cows, and claim to have succeeded in only 37 cases. In 1862, while stationed at Hilton Head, S. C., I had the opportunity of attempting this service. I covered the head of a six months old calf with a shirt saturated with variolous matter, and kept her breathing through it for 24 hours. I inoculated her in many places with matter in its most active stage, but failed to infect her. In Mr. Ceely's successful cases he continued the inoculation from heifer to heifer, and finally produced a virus with which he safely and protectively vaccinates the human animal.

Other experimentors have for the moment claimed success until painfully informed of their failure by the appearance of small-pox as the result of the use of their virus. Such an instance occurred in our own State last year.

Dr. Meyer, of Monee, a P. O. hamlet in Mattison, took matter from a heifer which he had succeeded in variolating, and introduced it into several families. The majority of his patients suffered a mild small-pox, but several developed the disease in a confluent form. The reporter adds, that the Doctor's Teutonic neighbors were looking for him, and anxious that he should decorate the bottom of a well, but he had left the bailiwick. I know of no other similar attempt in this country.

Dr. Martin says: "It is my conviction that in some mysterious manner nature may elaborate vaccinia from variola in the system of the milch cow, but that man can with no certainty repeat her process at will, nor does nature herself always accomplish it with success."

With the plentiful supply of pure vaccine matter now to be had in all civilized communities, it appears to me a criminal act to attempt this production of virus from human variola. Small-pox virus should never be touched but to destroy it. To attempt its cultivation, even though sometimes successful, resembles the proposition to cultivate tigers in a country for the purpose of destroying the wolves when these little animals become troublesome.

I conclude that we have four sources of vaccine supply now in popular use in this country:

1. The Beaugency stock introduced by Dr. Martin.
2. The Belgian stock introduced also by Dr. Martin, and by Dr. Griffin.
3. The culture of Dr. Griffiths, of Brooklyn, from a case of spontaneously appearing disease observed by him in the State of New York.
4. Humanized virus from all the above stocks.

